# Patterns of recurrent varicose veins after surgery (REVAS): A systematic review and network meta-analysis of randomized trials

**DOI:** 10.1177/02683555261418937

**Published:** 2026-01-23

**Authors:** Konstantinos Kavallieros, Adam M. Gwozdz, Benedict Turner, Giannis Konstantinou, Emmanuel Giannas, Iris Soteriou, Julianne Stoughton, Alun H. Davies

**Affiliations:** 1 Academic Section of Vascular Surgery, Department of Surgery and Cancer, 4615 Imperial College London, London, UK; 2 Faculty of Medicine, 4615 Imperial College London, London, UK; 3 Department of Vascular Surgery, 2348 Massachusetts General Hospital, Stoneham, MA, USA

**Keywords:** varicose veins, recurrence, endovenous ablation, REVAS

## Abstract

**Background:**

Recurrence of superficial venous incompetence is common following interventional treatment and a classification system (Recurrent Varices After Surgery, REVAS) has been developed. However, it is not known whether specific, predictable patterns of reflux occur following treatment nor how these may vary by treatment modality. This study aimed to explore varicose vein recurrence patterns according to procedural technique.

**Methods:**

Following PRISMA guidelines and a registered protocol (CRD42023455512), MEDLINE, Embase, and ClinTrials.gov were searched for randomized control trials (RCTs) on surgical or endovenous treatment of primary saphenous vein insufficiency with at least 1-year follow-up, and assessment of recurrence patterns. The primary outcome was reflux recurrence as per the REVAS classification. A random-effects network meta-analysis was conducted in R, calculating risk ratios and 95% confidence intervals (CIs).

**Results:**

The 3467 records identified yielded 23 unique RCTs, investigating 8 different modalities. Recurrence rates varied by anatomical section: saphenofemoral junction (SFJ) showed 23.6% cumulative recurrence, thigh perforators 7.6%, and lower leg perforators 4.7% recurrence. Endovenous laser ablation (EVLA) and foam sclerotherapy (FS) had higher risk of SFJ recurrence compared to HLS with a risk ratio of 2.29 (1.40–3.76) and 2.09 (1.20–3.62) (I^2^ = 47.7%). EVLA was associated with a reduced risk of thigh perforator recurrence compared to HLS (0.45, (0.21–0.93)) (I^2^ = 0%). FS was associated with higher risk of recanalization compared to HLS (4.05 (2.23–7.35)), and EVLA (3.14 (1.82–5.41)), Both EVLA and FS were associated with lower risk of neovascularization, compared to HLS; 0.28 (0.18–0.43) and 0.18 (0.08–0.40), respectively (I^2^ = 0%).

**Conclusion:**

Recurrence patterns varied by treatment modality, with HLS showing lower SFJ and ASV recurrence, while endovenous methods had less neovascularization and thigh perforator recurrence. Concerningly, only 13% of RCTs reported recurrence using REVAS. Improved reporting of varicose vein recurrence to delineate reflux sources will allow better technical outcome assessment and enhanced patient care.

## Background

Superficial venous insufficiency is highly prevalent in the population with approximately 15% of males and 25% of females experiencing lower-extremity venous insufficiency.^1,2^ This most commonly presents as varicose veins but may also include leg pain, swelling or venous ulceration. The health-related quality of life (HRQOL) of individuals with chronic venous insufficiency can be substantially impaired.^3^ However, treating axial reflux and tributary varicose veins can alleviate the symptoms of pain, swelling, and discomfort after prolonged standing, whilst potentially reducing associated complications.^4^ Despite advancements in preoperative evaluation and treatment methods, recurrence rates following varicose vein procedures vary widely, ranging from 20% to 80% of cases.^5–7^

The traditional gold standard for treating varicosities of the great saphenous veins (GSV) used to involve high ligation at the saphenofemoral junction (SFJ), followed by stripping (HLS). However, with the development of endovenous techniques, guidelines from various professional bodies are now recommending endovenous ablation as the first-line therapy for symptomatic varicose veins.^4,8^

Recurrence of varicose veins is a crucial outcome measure for evaluating treatment effectiveness. Recurrence rates are well-documented, and a classification system known as REVAS (recurrent varices after surgery) has been developed, encompassing anatomic and clinical recurrence categorized by site, cause, and anatomy.^9^ However, specific sources of reflux following intervention remain unclear; for example there may be differences in the patterns of recurrence according to different treatment modalities and the presence or absence of co-interventions such as phlebectomies. To address this gap, the present study employs a network meta-analysis to investigate varicose vein recurrence patterns by procedural technique.

## Methods

### Study design

This study was conducted in accordance with a protocol agreed upon by all the authors and the Preferred Reporting Items for Systematic Reviews and Meta-Analysis (PRISMA) guidelines.^10^ The study’s protocol was registered prospectively with PROSPERO (registration number: CRD42023455512).

### Search strategy

The databases MEDLINE (PubMed) and EMBASE were searched using the search engine Ovid, without any restrictions regarding study design, date or language. The clinical trials registry ClinicalTrials.gov was also searched for relevant studies. A focused systematic review of the literature was performed under the guidance of a qualified medical librarian to ensure a robust search strategy. The last day of the search was in October 2023. A combination of MeSH terms and keywords were used to produce the search strategy. Details regarding the search strategy are available in the Appendix I.

### Eligibility criteria

Studies reporting on outcomes following the use of any of the following therapies for lower extremity, primary, superficial venous insufficiency, targeted to at least one of, Great Saphenous vein (GSV) or Small Saphenous Vein (SSV): Any open surgical (e.g., high stripping and ligation (HLS), cryostripping) or endovenous (e.g., Radiofrequency Ablation (RFA), Endovenous Laser Ablation (EVLA), Foam Sclerotherapy (FS), mechanochemical ablation (MOCA), and CHIVA) were considered.

Only randomized control trials (RCTs), comparing at least two different modalities were included. A minimum follow-up of 1 year, DUS assessment at follow-up, and reporting on at least one outcome of interest was also required. Prospective non-randomized studies as well as retrospective studies were not considered. Case reviews, case studies, reviews, editorials, expert consensus documents, and abstracts were excluded. Non-human studies as well as those not in English were not considered

### Data extraction

Two independent reviewers (K.K, G.K) performed title and abstract screening using Covidence (Melbourne, Australia). Full-text review was performed by two independent reviewers (K.K, G.K, E.G, I.S) and any disagreements were discussed with senior authors (B.T., A.M.G, J.S), until a consensus was reached. A data charting spreadsheet was developed using Microsoft Excel (Redmond, Washington, USA) and was populated with (1) study demographics (author, year of publication, country of publication, study design) (2) participant demographics (age, gender, CEAP classification), (3) treatment type, (4) treatment indication, (5) anatomical location of treatment (6) numbers treated (7) numbers with overall recurrence and (8) numbers with each specific pattern of recurrence as per the REVAS classification.^9^ The outcomes for each intervention group were recorded separately. Crude event rates were copied from the studies, for each of the outcomes.

### Outcomes

The primary outcome was recurrence pattern as per the REVAS classification system. The classification defines reflux by the source of recurrence (sapheno-femoral junction (SFJ), sapheno-popliteal junction (SPF) or thigh/lower leg perforators), the nature of recurrence (recanalization or neovascularization), and the contribution from persistent saphenous vein trunks (anterior saphenous vein (ASV), small saphenous vein (SSV), and above knee (AK) or below-knee (BK) GSV).

A recanalized saphenous vein was defined as an open part of the treated vein segment. Neovascularization was defined as serpentine vessels emanating from the SFJ that were not present on early duplex imaging.

### Data and statistical analysis

Demographic characteristics were summarized using descriptive statistics, number (%) and median (IQR). Cumulative rates of recurrence were summarized using number (%). A random-effects network meta-analysis was conducted in R (R 4.3.2 GUI, Mac OS), calculating risk ratios and 95% confidence intervals (CIs). Continuity correction was achieved by adding 0.1 to all outcomes reporting 0 events. R packages “netmeta”, “rmeta”, “readxl”, “getmc”, “tidyverse” and “ggplot2” were utilized. Results were considered statistically significant at the *p* < 0.05 level if the 95% CI did not include the value of 1.

### Risk of bias

Two independent reviewers (K.K, I.S) performed a risk of bias assessment using the Cochrane Risk of Bias quality assessment tool.^11^

## Results

**Figure 1.**
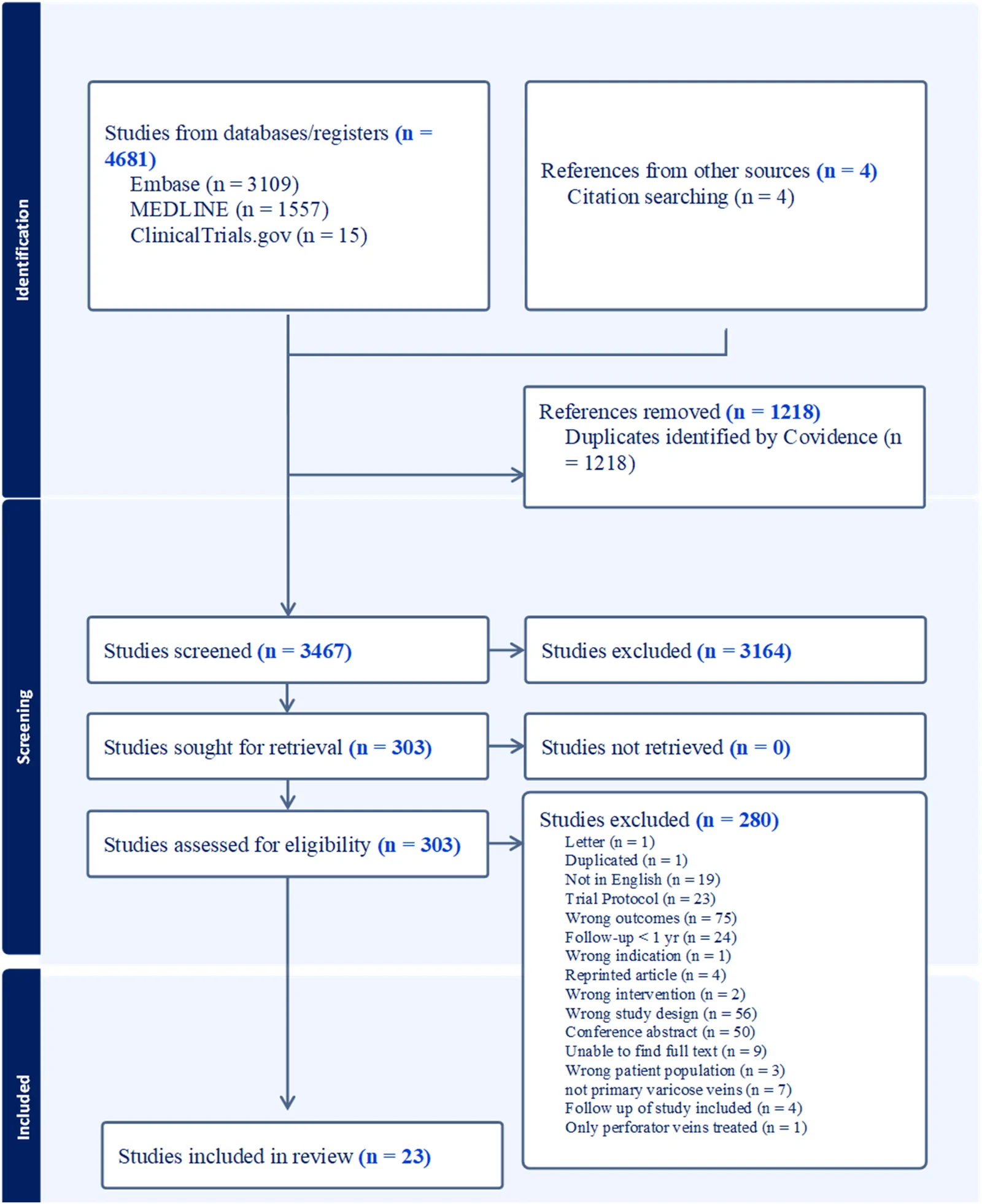
PRISMA flow diagram source of recurrence.

In total, 3467 studies were screened, with 303 studies being retrieved for full text review; 23 were included (Figure 1).^12–34^ The RCTs included were published between 1999 and 2022, with the majority being from centers in Europe (20/23); two were from centers in the USA, and one from China. The median average age of included participants was 49 years (IQR: 47.7–52); the majority were female (63.6%). Most participants included in the studies had C2 or C3 disease on the CEAP classification (50.1% and 30.7%, respectively); 13.0% had C4 disease and only 1.89%, 2.47% and 1.80% of patients had C1, C5 and C6, disease, respectively.

**Figure 2.**
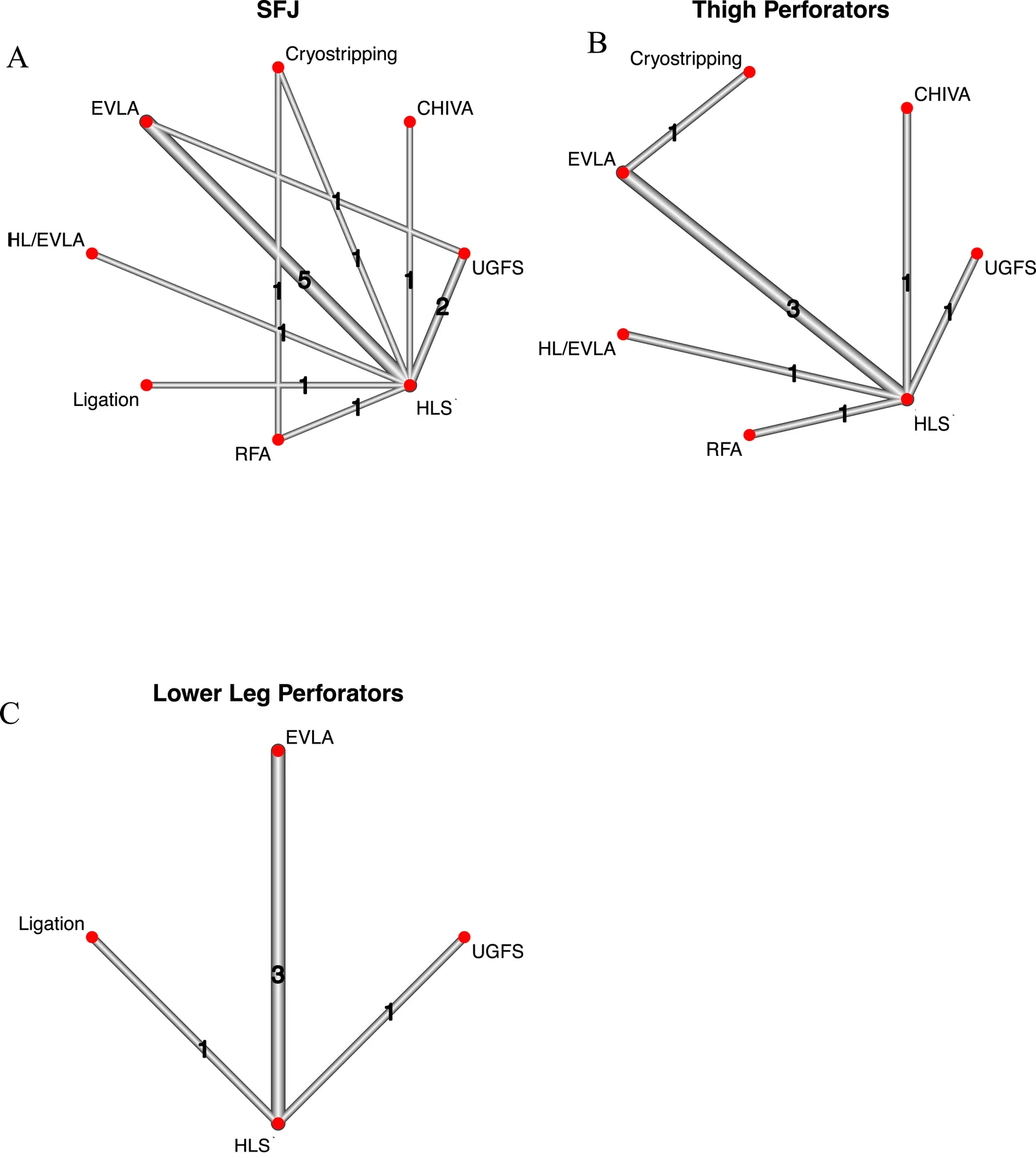
Network graphs for source of recurrence outcomes. (a) Pairwise comparisons for SFJ recurrence (*n* = 14), (b) pairwise comparisons comparisons for thigh perforator recurrence (*n* = 8), (c) pairwise comparisons for lower leg perforators (*n* = 5). Recurrence at the sapheno-popliteal junction and from popliteal perforators was only reported in one RCT and hence is not included here.

Studies consisted of 23 randomized trials, with 51 trial arms (Supplemental Table 1, for further study data); these included 3279 limbs at follow-up. The trials investigated a total of 8 different modalities (Figure 2). Surgical management with HLS was the most investigated modality (20/23 RCTs); EVLA was examined in 69.5% (16/23 RCTs); FS, CHIVA, MOCA, RFA and cryostripping were less frequently. The majority of studies examined treatment of the great saphenous vein (GSV) in isolation. One study treated both GSV and SSV, and one examined the small saphenous vein (SSV) alone (this was excluded from the quantitative network meta-analysis).^24^ The anterior saphenous vein (ASV) was not the primary treatment target of any of the studies. Of the included studies, 13% (3/23) reported recurrence using REVAS (source of recurrence, nature of source and contribution from persistent saphenous trunk). The overall recurrence rates according to procedure were summarised by a 2022 network meta-analysis of RCTs – this data was therefore not collected, given the exclusion of RCTs reporting general recurrence but no patterns of recurrence.^35^

### Source of recurrence

Thirteen studies described source of recurrence.^12,15,17–20,23,25,28–30,32,33^ The total number of analyzed legs was 1722. The number of studies contributing to each direct pairwise comparisons for Sources of recurrence, is shown in the network graphs (Figure 2(a)–(c)). Only two studies reported on recurrence from the SPJ or popliteal perforators and these outcomes were therefore not included in the network meta-analysis. The commonest source of recurrence was the SFJ: 23.6% recurrence rate (350 events, 1484 legs, 10 studies). Recurrence via thigh perforators was less common (7.6% recurrence rate) (88 events, 1156 legs, 8 studies); lower leg perforators were the source in 4.7% of legs (45 events, 966 legs, 5 studies).

**Figure 3.**
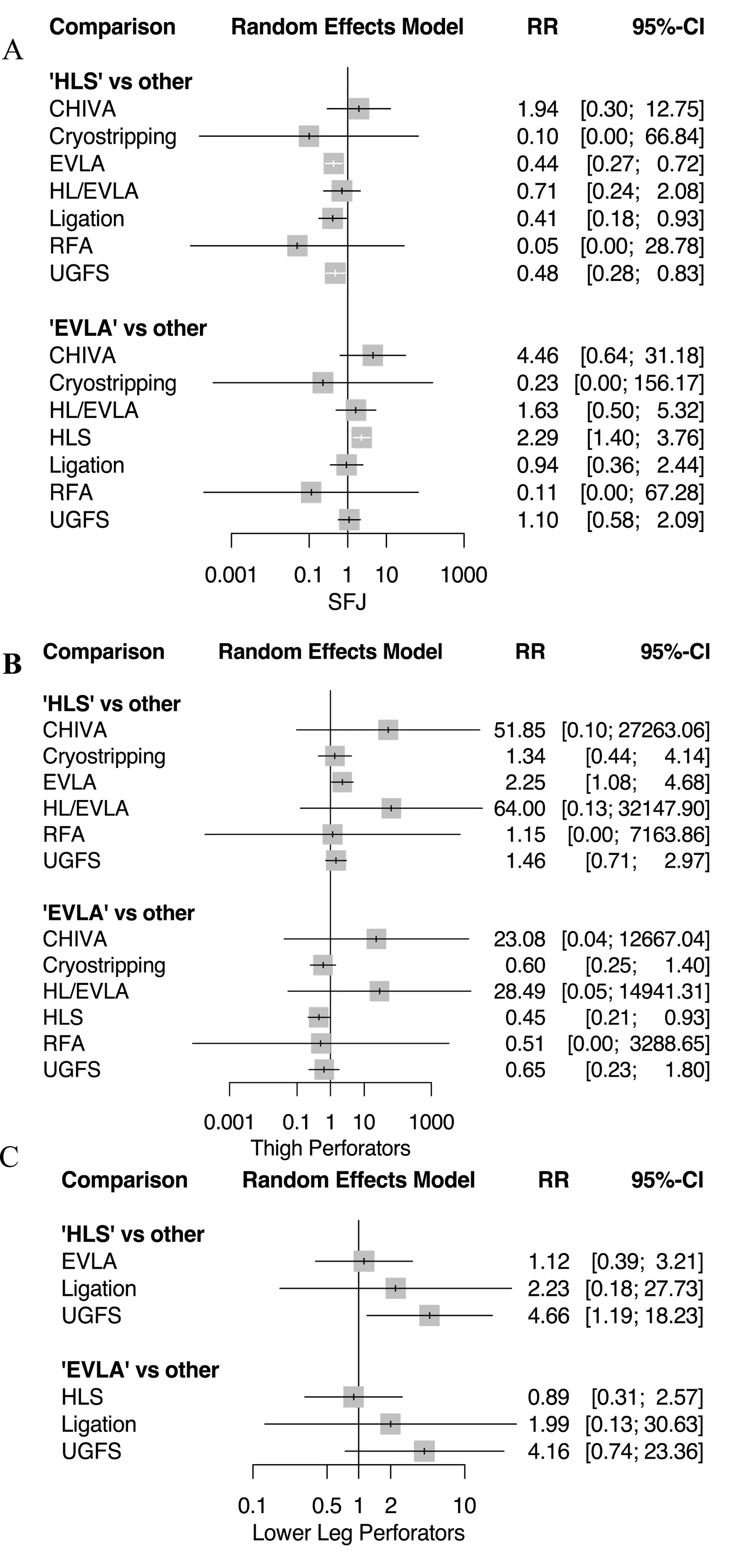
Forest plots for network meta-analysis of sources of recurrence.

Forest plots for the network meta-analysis for rates of recurrence through the various sources are shown in Figure 3(a)–(c). EVLA and FS had higher risk of SFJ recurrence compared to HLS with risk ratios (RRs) of 2.29 (1.40–3.76) and 2.09 (1.20–3.62), respectively (I^2^ = 47.7%). EVLA was associated with a reduced risk of thigh perforator recurrence compared to HLS (0.45, (0.21–0.93)) (I^2^ = 0). EVLA had similar risk of recurrence through the SFJ, thigh perforators and lower leg perforators compared to CHIVA and RFA. Supplemental Tables II–IV (Appendix) show league tables displaying RRs and 95% CI from all possible comparisons.

### Nature of recurrence

**Figure 4.**
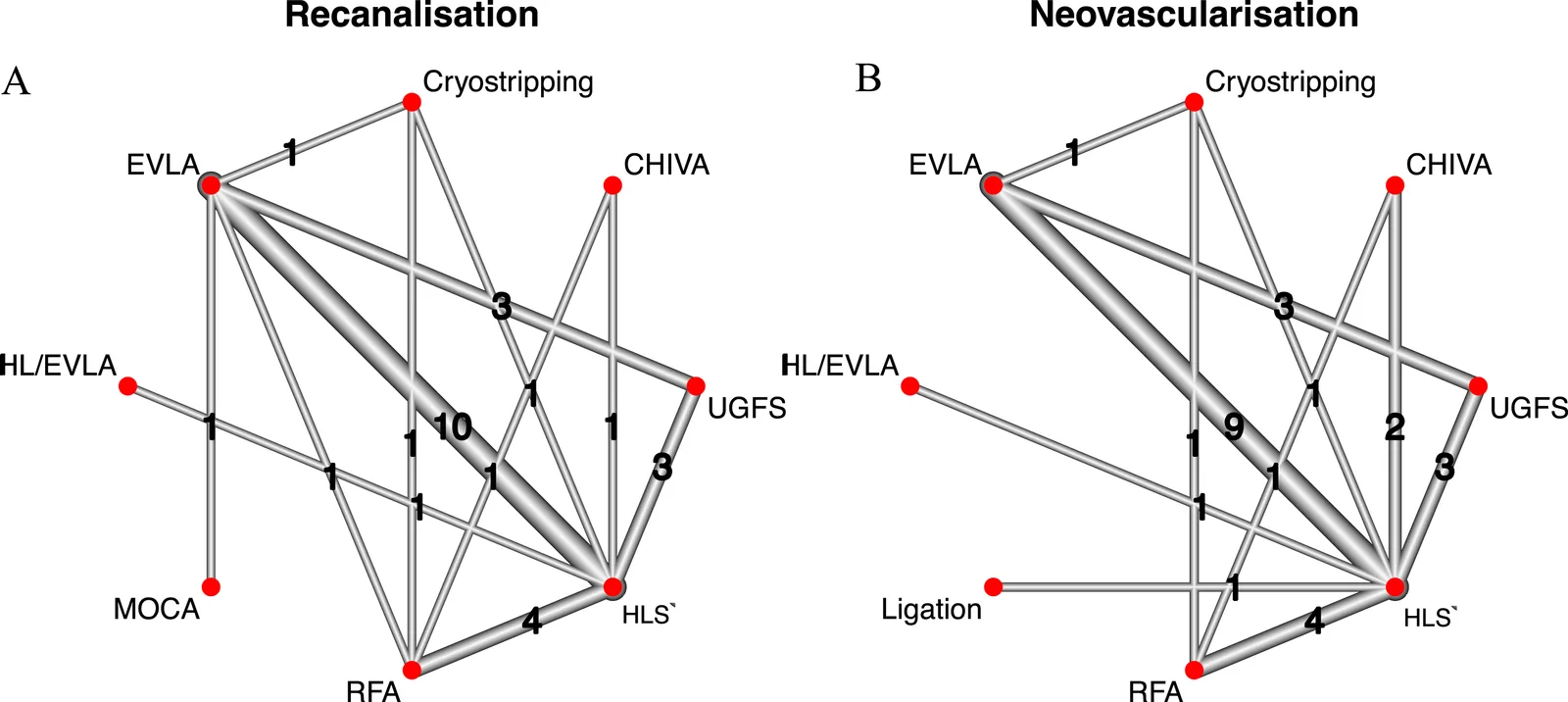
Network graphs for nature of recurrence outcomes. (a) Pairwise comparisons for recanalization (*n* = 28), (b) pairwise comparisons for neovascularization (*n* = 27).

Twenty studies of 3025 legs described nature of recurrence (i.e., recanalization or neovascularization).^12–16,18–23,25,27–34^ The number of studies contributing to each direct pairwise comparisons for nature of recurrence outcomes, is shown in the network graphs (Figure 4(a) and (b)). Recanalization was more common than neovascularization (12.0% (324 events, 2692 legs, 18 studies) versus 9.9% (239 events, 2410 legs, 17 studies).

**Figure 5.**
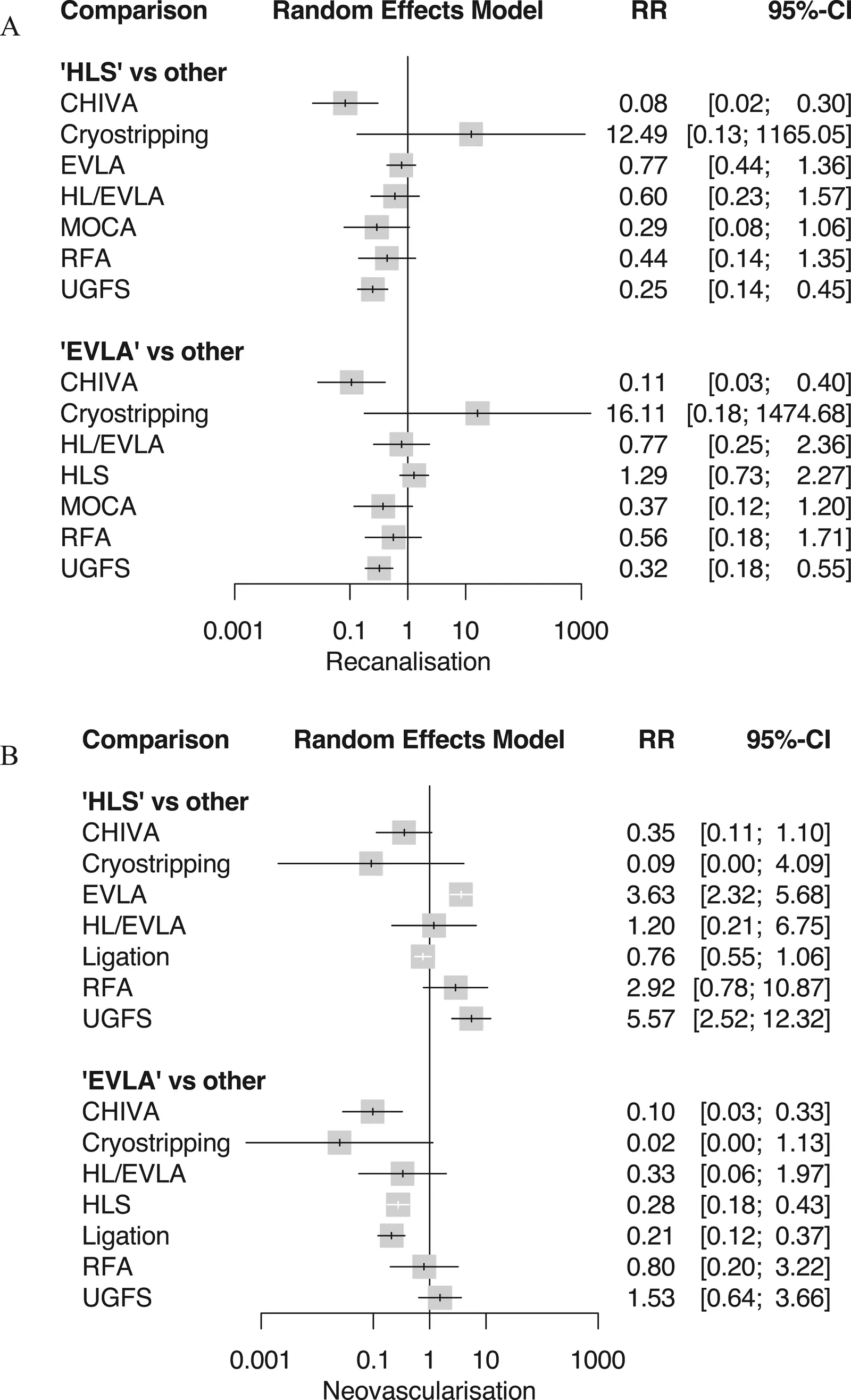
Forest plots for network meta-analysis of nature of recurrence.

Forest plots for the network meta-analysis are shown in Figure 5(a) and (b). FS was associated with higher risk of recanalization compared to HLS (4.05 (2.23–7.35)), and EVLA (3.14 (1.82–5.41)), (I^2^ = 25.8%). Both EVLA and FS were associated with lower risk of neovascularization, compared to HLS; 0.28 (0.18–0.43) and 0.18 (0.08–0.40), respectively (I^2^ = 0%). EVLA was associated with a similar risk of recanalization compared to RFA and MOCA. In addition, EVLA showed lower risk of both recanalization and neovascularization compared to CHIVA, with risk ratios of 0.11 (0.03–0.40) and 0.10 (0.03–0.33) respectively. Supplemental Tables V and VI (Appendix), show league tables displaying RRs and 95% CI from all possible comparisons.

### Contribution from persistent saphenous trunk

**Figure 6.**
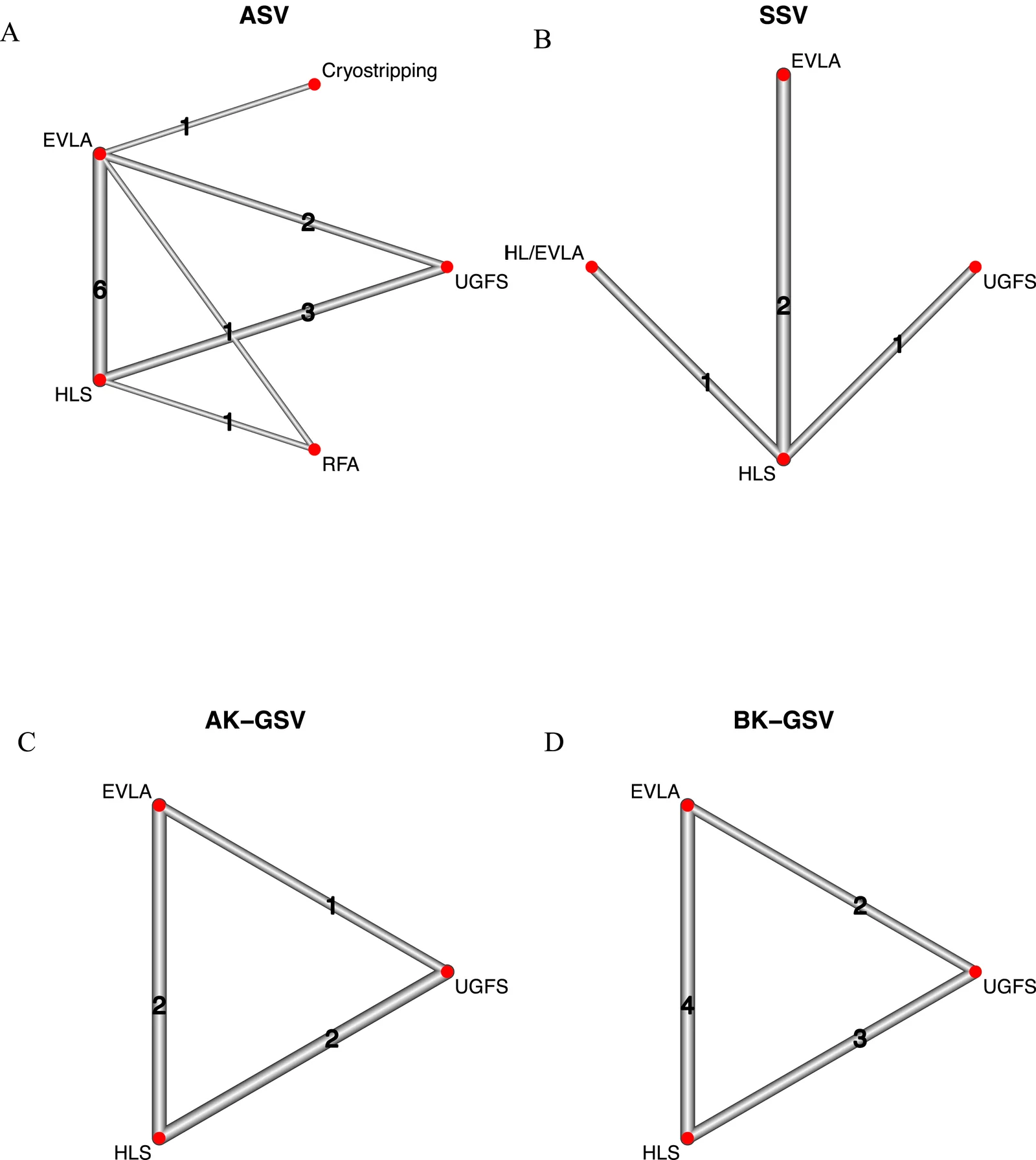
Network graphs for contribution of saphenous trunks. (a) Pairwise comparisons for AASV (*n* = 14), (b) pairwise comparisons for SSV (*n* = 4), (c) pairwise comparisons for AK-GSV (*n* = 5), (d) pairwise comparisons for BK-GSV (*n* = 9).

Twelve studies described recurrence through persisting saphenous trunks.^12,15–20,23,25,29,30,34^ The total number of analyzed legs was 1841. The number of studies contributing to each direct pairwise comparisons is shown in the network graphs (Figure 6(a)–(d)). The most often reported recurrence was through the anterior saphenous vein (ASV), with 9.8% recurrence rate (146 events, 1488 legs, 10 studies). BK-GSV recurrence was reported in five studies, with a cumulative rate of 15.4% (149 events, 966 legs, 5 studies); recurrence of the AK-GSV was reported in three studies with 24.5% recurrence (171 events, 697 legs, 3 studies). Recurrence of the SSV was reported in four studies with 24.5% recurrence 3.8% (30 events, 794 legs, 4 studies).

**Figure 7.**
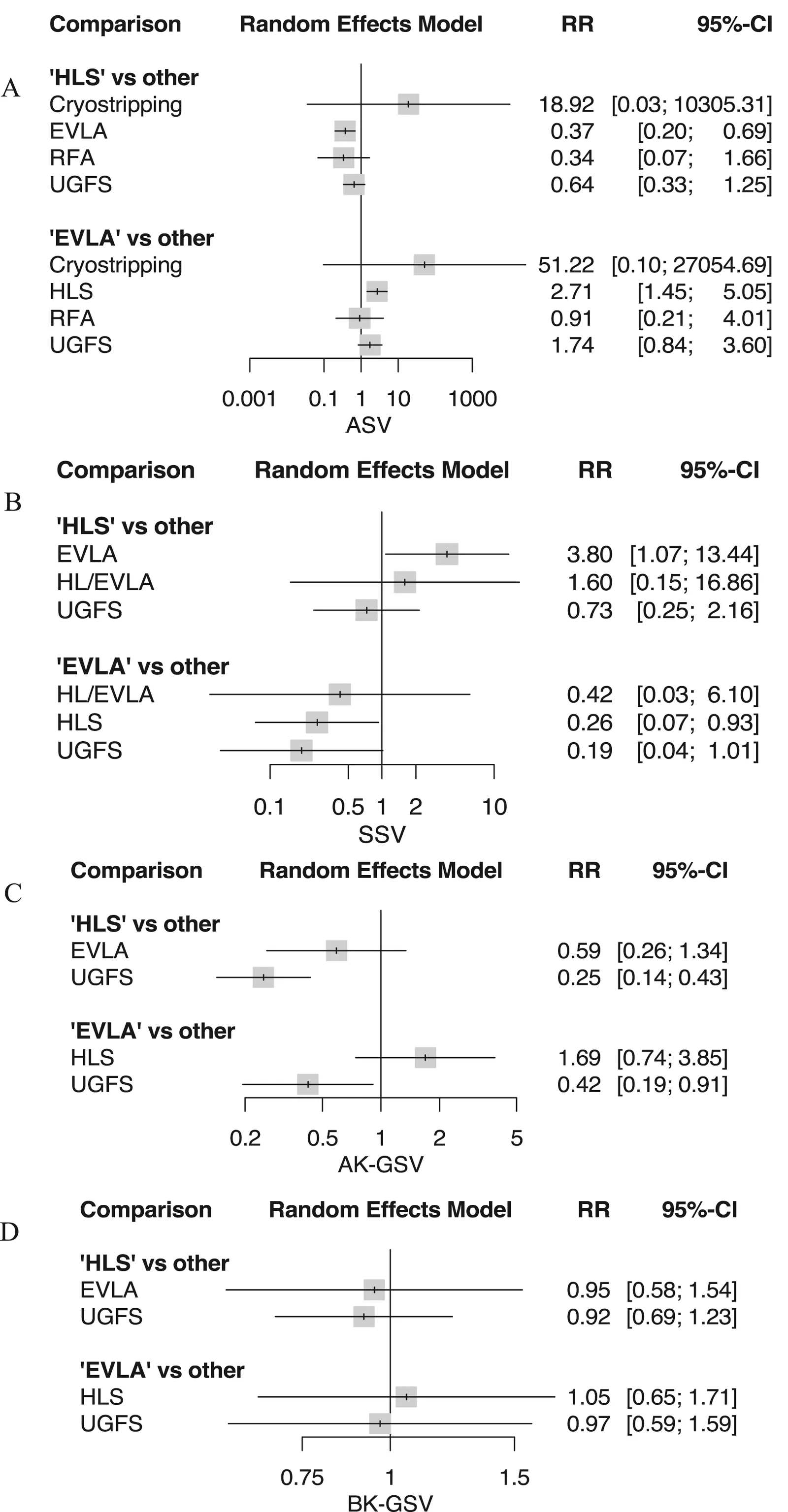
Forest plots for network meta-analysis of recurrence through contributory saphenous trunks.

Forest plots for the network meta-analysis are shown in Figure 7(a)–(d). EVLA was associated with greater risk of recurrence through the ASV compared to HLS (2.71 (1.45–5.05)) (I^2^ = 19.6%). HLS and EVLA were associated with decreased risk of AK-GSV recurrence, compared to FS (0.25 (0.14–0.43) and 0.42 (0.19–0.91), respectively) (I^2^ = 39.3%). Supplemental Tables VII–X (Appendix), show league tables displaying RRs and 95% CI from all possible comparisons.

### Methodological quality of included studies

The majority of the studies included in this analysis were assessed as having adequate methodological quality, as detailed in Supplemental Table XI (Appendix). Most of these studies implemented random sequence generation. The risk of performance and detection bias was evaluated as moderate to high for subjective assessments related to patterns of recurrence. Despite the difficulty in blinding of patients and assessors, the evaluation of procedural success and recurrence, being objective DUS measures, was considered to have an overall moderate risk of bias.

## Discussion

This systematic review and network meta-analysis aimed to elucidate the rates of different patterns of recurrence of varicose veins (VV) based on evidence from 23 randomised clinical trials. Our study builds on the findings of a 2022 network meta-analysis,^35^ which reported overall recurrence rates among RCTs investigating different interventions for varicose veins. Their findings suggest that although cyanoacrylate embolization (CAE) offers the lowest risk of initial procedural failure, HLS resulted in lower rates of long-term recurrence without significantly increasing morbidity compared to other endovenous options. Our findings contribute to the understanding of the efficacy of various treatment modalities for VV recurrence, by shedding light on the patterns of recurrence following each approach.

With a recognition of the need to combine imaging and clinical diagnosis to identify the origin of recurrent varicose veins, Perrin et al. in 1998, developed the REVAS classification, to supplement the CEAP.^5,9^ Our systematic review attempted to follow this classification; this was first done in 2016 by O’Donnell et al.^5^ However, in light of more studies being published since then, we have utilized a network meta-analysis to provide direct comparisons for patterns of REVAS.

As expected, the SFJ was the source that exhibited the highest recurrence rate across interventions. Endovenous methods such as EVLA and FS demonstrate higher risk of SFJ recurrence compared to surgical management, an observation which has been repeatedly confirmed. Conversely, EVLA was associated with a reduced risk of thigh perforator recurrence compared to HLS. This may suggest its superiority in mitigating reflux in the thigh, or it may be explainable by phlebectomies that are often performed contemporaneously. The use of additional phlebectomies was inconsistently reported and was therefore challenging to account for in the meta-analysis. Low rates of recurrence were observed through the SPJ and through lower leg perforators.

The nature of recurrence was the outcome most often reported. Most studies assessed and reported on the occurrence of target vein recanalization, or neovascularization. Neovascularization has long been implicated as one of the leading mechanisms leading to VV recurrence.^5,7,9,36–38^ It is thought to be the result of angiogenesis secondary to trauma to the tissue following surgical dissection^38,39^; extra-venous inflammation is, believed, however, not to occur following EVLA. It therefore follows that EVLA may not promote angiogenesis at the operative site.^40^ Our findings support this hypothesis, showing that HLS is associated with an approximately threefold increase in the rate of neovascularization compared to endovenous treatment methods.

Furthermore, contribution to recurrence of persistent saphenous trunks, particularly the ASV, warrant attention in treatment planning and outcome assessment. EVLA appears to be associated with a greater risk of recurrence through the ASV compared to HLS. The role of the ASV in primary venous insufficiency is currently widely debated, with questions as to its anatomical significance as well as its potential to cause recurrence.^41–43^ The SYNCHRONOUS-Study, an ongoing RCT investigating EVLA with or without concurrent ASV ablation will provide evidence to further inform the debate surrounding the increasingly recognized significance of the ASV.^44^ Findings regarding the AK-GSV and BK-GSV are limited by their scarce reporting in RCTs. However, the observed 15.4% recurrence through the BK-GSV is notable. Because none of the included studies treated the full below-knee segment, this recurrence is more likely attributable to new reflux or varicosities arising from an untreated portion rather than recanalization of a treated segment. While our analysis cannot determine the precise mechanism, the finding adds weight to the current discussion regarding whether complete treatment of a refluxing BK-GSV should be considered at the initial intervention.

An important limitation of the study was that only a small proportion of the published trials on varicose vein management reported on any patterns of recurrence, and of those that did, a very small proportion (13%) reported recurrence using the REVAS classification system. Hence the findings presented in the network meta-analysis have been sampled from a minority of studies in the literature – this could be an important source of selection bias. This lack of standardized reporting of patterns is also undoubtably an important source of heterogeneity in the results. Outcomes were extracted only if reported explicitly in text. Cumulative recurrence at each topographical site (e.g., thigh, popliteal fossa, calf) irrespective of source was seldom explicitly reported, and thus not presented. This highlights the need for improved reporting standards to facilitate accurate assessment of technical outcomes and guide clinical decision-making. Furthermore, despite implementing a minimum follow-up of 1 year as an inclusion criterion, the grouping of trials irrespective of length of follow-up period in the same analysis is another limitation. The small number of studies, however, made further sub-grouping based on follow-up period challenging. A further limitation of our study is the disproportionate representation of HLS among the included RCTs, reflecting historical practice patterns rather than contemporary treatment use in many countries. This is compounded by the exclusion of cyanoacrylate closure, as published RCTs predominantly report occlusion success without providing the recurrence-pattern data required for this analysis. Nevertheless, understanding recurrence pathways across all modalities—including those less frequently performed today—remains valuable, as it provides updated quantitative benchmarks and highlights anatomical routes of failure that continue to inform clinical decision-making and future trial design.

## Conclusion

In conclusion, our study contributes to a deeper understanding of varicose vein recurrence patterns and associated complications following different treatment modalities for SVI. Delineating the sources of reflux and highlighting the efficacy and limitations of each intervention can pave the way for improved treatment strategies and enhanced patient care in the management of varicose veins. However, it is limited by the scarce reporting of patterns of REVAS in the wider varicose vein literature. Further research focusing on long-term outcomes and standardized reporting of recurrence patterns is warranted to refine treatment algorithms and optimize patient outcomes in the management of SVI.

## Supplemental material


Supplemental Material - Patterns of recurrent varicose veins after surgery (REVAS): A systematic review and network meta-analysis of randomized trials
Supplemental Material for Patterns of recurrent varicose veins after surgery (REVAS): A systematic review and network meta-analysis of randomized trials by Konstantinos Kavallieros, Adam M. Gwozdz, Benedict Turner, Giannis Konstantinou, Emmanuel Giannas, Iris Soteriou, Julianne Stoughton, Alun H. Davies in Phlebology
