# Supplementary material for: Patterns of recurrent varicose veins after surgery (REVAS): A systematic review and network meta-analysis of randomized trials
Source: Phlebology. 2026 Jan 23;41(8):658–68. doi: 10.1177/02683555261418937 (PMC13490677; doi:10.1177/02683555261418937)
Supplement: Supplementary file 1 [file sj-pdf-1-phl-10.1177_02683555261418937.pdf]

Supplemental Table 1 (Online only): Summary characteristics of included studies

**Author (year)**	*Country*	*Study Arms*	*Interventions*	*Sample size (Limbs)*	*Follow-up (years)*
**Biemans (2013)**	*Netherlands*	*3*	*EVLA vs UGFS vs HLS*	*240*	*1*
**Canas (2021)**	*Spain*	*3*	*RFA vs HLS vs CHIVA*	*225*	*2*
**Carandina (2008)**	*Italy*	*2*	*HLS vs CHIVA*	*150*	*10*
**Christenson (2010)**	*Switzerland*	*2*	*EVLA vs HLS*	*204*	*2*
**Darwood (2008)**	*UK*	*2*	*EVALA vs HLS*	*136*	*1*
**Disselhoff (2011)**	*Netherlands*	*2*	*EVLA vs Cryostripping*	*120*	*5*
**Dwerryhouse (1999)**	*UK*	*2*	*HLS vs Ligation*	*133*	*5*

**Eggen (2022)**	*Netherlands*	*2*	*HLS vs EVLA*	*130*	*10*
**Kalteis (2015)**	*USA*	*2*	*HLS vs HL/EVLA*	*100*	*5*
**Kempeneers (2022)**	*Belgium*	*2*	*EVLA vs RFA*	*280*	*1*
**Lam (2018)**	*Netherlands*	*2*	*UGFS vs HLS*	*460*	*8*
**Lurie (2005)**	*USA*	*2*	*RFA vs HLS*	*86*	*2*
**Mohamed (2021)**	*UK*	*2*	*EVLA vs MOCA*	*150*	*1*
**Perala (2005)**	*Finland*	*2*	*RFA vs HLS*	*28*	*3*
**Pronk (2010)**	*Netherlands*	*2*	*HLS vs EVLA*	*130*	*1*
**Rasmussen (2013)**	*Denmark*	*2*	*HLS vs EVLA*	*137*	*5*
**Rass (2015)**	*Germany*	*2*	*EVLA vs HLS*	*400*	*5*

**Samuel (2013)**	*UK*	*2*	*EVLA vs HLS*	*106*	*5*
**Stotter (2006)**	*Germany*	*3*	*RFA vs HLS vs Cryostripping*	*60*	*1*
**Vahaaho (2017)**	*Finland*	*3*	*HLS vs EVLA vs UGFS*	*196*	*5*
**Van der Velden (2015)**	*Belgium*	*3*	*HLS vs EVLA vs UGFs*	*224*	*5*
**Wallace (2018)**	*UK*	*2*	*HLS vs EVLA*	*280*	*5*
**Yang (2013)**	*China*	*2*	*EVLA + ligation vs HLS*	*206*	*2*

Supplementary Table II: Network meta-analysis league table for SFJ recurrence.							
**CHIVA**							
0.05 (0.00 to 44.87)	**Cryostripping**						
0.22 (0.03 to 1.57)	4.36 (0.01 to 2970.58)	**EVLA**					
0.37 (0.04 to 3.19)	7.11 (0.01 to 5190.34)	1.63 (0.50 to 5.32)	**HL/EVLA**				
0.51 (0.08 to 3.37)	10.00 (0.01 to 6683.98)	2.29 (1.40 to 3.76)	1.41 (0.48 to 4.11)	**HLS**			
0.21 (0.03 to 1.63)	4.08 (0.01 to 2871.41)	0.94 (0.36 to 2.44)	0.57 (0.15 to 2.22)	0.41 (0.18 to 0.93)	**Ligation**		
0.03 (0.00 to 19.44)	0.50 (0.04 to 5.58)	0.11 (0.00 to 67.28)	0.07 (0.00 to 44.29)	0.05 (0.00 to 28.78)	0.12 (0.00 to 74.35)	**RFA**	
0.25 (0.03 to 1.75)	4.79 (0.01 to 3277.56)	1.10 (0.58 to 2.09)	0.67 (0.20 to 2.25)	0.48 (0.28 to 0.83)	1.17 (0.44 to 3.15)	9.58 (0.02 to 5648.79)	**UGFS**

Supplementary Table III: Network meta-analysis league table for Thigh Perforator recurrence.						
**CHIVA**						
0.03 (0.00 to 15.05)	**Cryostripping**					
0.04 (0.00 to 23.77)	1.67 (0.71 to 3.93)	**EVLA**				
1.23 (0.00 to 8418.23)	47.68 (0.09 to 26496.27)	28.49 (0.05 to 14941.31)	**HL/EVLA**			
0.02 (0.00 to 10.14)	0.74 (0.24 to 2.30)	0.45 (0.21 to 0.93)	0.02 (0.00 to 7.85)	**HLS**		
0.02 (0.00 to 1035.91)	0.86 (0.00 to 5736.75)	0.51 (0.00 to 3288.65)	0.02 (0.00 to 817.28)	1.15 (0.00 to 7163.86)	**RFA**	
0.03 (0.00 to 15.37)	1.08 (0.29 to 4.11)	0.65 (0.23 to 1.80)	0.02 (0.00 to 11.90)	1.46 (0.71 to 2.97)	1.26 (0.00 to 8066.55)	**UGFS**

Supplementary Table IV: Network meta-analysis league table for Lower Leg Perforator recurrence.			
**EVLA**			
0.89 (0.31 to 2.57)	**HLS**		
1.99 (0.13 to 30.63)	2.23 (0.18 to 27.73)	**Ligation**	
4.16 (0.74 to 23.36)	4.66 (1.19 to 18.23)	2.09 (0.12 to 36.68)	**UGFS**

Supplementary Table V: Network meta-analysis league table for Recanalization.							
**CHIVA**							
151.43 (1.49 to 15412.34)	**Cryostripping**						
9.40 (2.47 to 35.71)	0.06 (0.00 to 5.68)	**EVLA**					
7.28 (1.44 to 36.71)	0.05 (0.00 to 4.96)	0.77 (0.25 to 2.36)	**HL/EVLA**				
12.13 (3.30 to 44.64)	0.08 (0.00 to 7.47)	1.29 (0.73 to 2.27)	1.67 (0.64 to 4.35)	**HLS**			
3.52 (0.60 to 20.66)	0.02 (0.00 to 2.47)	0.37 (0.12 to 1.20)	0.48 (0.10 to 2.42)	0.29 (0.08 to 1.06)	**MOCA**		
5.28 (1.67 to 16.72)	0.03 (0.00 to 3.21)	0.56 (0.18 to 1.71)	0.73 (0.16 to 3.21)	0.44 (0.14 to 1.35)	1.50 (0.30 to 7.48)	**RFA**	
3.00 (0.75 to 11.95)	0.02 (0.00 to 1.86)	0.32 (0.18 to 0.55)	0.41 (0.13 to 1.27)	0.25 (0.14 to 0.45)	0.85 (0.24 to 3.06)	0.57 (0.17 to 1.87)	**UGFS**

Supplementary Table VI: Network meta-analysis league table for Neovascularization.							
**CHIVA**							
0.26 (0.00 to 13.23)	**Cryostripping**						
10.29 (3.04 to 34.76)	40.05 (0.88 to 1818.85)	**EVLA**					
3.40 (0.43 to 26.82)	13.23 (0.20 to 867.02)	0.33 (0.06 to 1.97)	**HL/EVLA**				
2.83 (0.91 to 8.79)	11.02 (0.24 to 497.32)	0.28 (0.18 to 0.43)	0.83 (0.15 to 4.69)	**HLS**			
2.16 (0.66 to 7.03)	8.41 (0.18 to 384.98)	0.21 (0.12 to 0.37)	0.64 (0.11 to 3.69)	0.76 (0.55 to 1.06)	**Ligation**		
8.26 (1.88 to 36.32)	32.15 (0.66 to 1576.12)	0.80 (0.20 to 3.22)	2.43 (0.28 to 21.32)	2.92 (0.78 to 10.87)	3.82 (0.98 to 14.84)	**RFA**	
15.76 (3.95 to 62.89)	61.37 (1.26 to 2994.68)	1.53 (0.64 to 3.66)	4.64 (0.69 to 31.07)	5.57 (2.52 to 12.32)	7.30 (3.09 to 17.24)	1.91 (0.41 to 8.88)	**UGFS**

Supplementary Table VII: Network meta-analysis league table for ASV.				
**Cryostripping**				
0.02 (0.00 to 10.31)	**EVLA**			
0.05 (0.00 to 28.80)	2.71 (1.45 to 5.05)	**HLS**		
0.02 (0.00 to 11.18)	0.91 (0.21 to 4.01)	0.34 (0.07 to 1.66)	**RFA**	
0.03 (0.00 to 18.71)	1.74 (0.84 to 3.60)	0.64 (0.33 to 1.25)	1.91 (0.37 to 9.86)	**UGFS**

Supplementary Table VIII: Network meta-analysis league table for AK-GSV.		
**EVLA**		
1.69 (0.74 to 3.85)	**HLS**	
0.42 (0.19 to 0.91)	0.25 (0.14 to 0.43)	**UGFS**

Supplementary Table IX: Network meta-analysis league table for BK-GSV.		
**EVLA**		
1.05 (0.65 to 1.71)	**HLS**	
0.97 (0.59 to 1.59)	0.92 (0.69 to 1.23)	**UGFS**

Supplementary Table X: Network meta-analysis league table for SSV.			
**EVLA**			
0.42 (0.03 to 6.10)	**HL/EVLA**		
0.26 (0.07 to 0.93)	0.62 (0.06 to 6.59)	**HLS**	
0.19 (0.04 to 1.01)	0.46 (0.03 to 6.09)	0.73 (0.25 to 2.16)	**UGFS**

Supplementary Table XI: Risk of Bias of included studies, using Cochrane risk of bias assessment tool

Trial	**Random Sequence Generation**	**Allocation Concealment**	**Blinding Participants and Personnel**	**Blinding of Outcome Assessment**	**Incomplete Outcome Data**	**Selective Reporting**	**Other: Outcomes clearly defined**	**Other: Inclusion/ Exclusion criteria clearly defined**	**Other: Surgeons sufficiently experienced in each procedure**
Biemans (2013)	+	+	+	+	+	+	+	+	+
Canas (2021)	+	+	-	-	+	+	+	+	+
Christenson (2010)	+	?	?	+	+	+	+	+	+
Carandina (2008)	+	+	-	+	+	+	+	+	+
Disselhoff (2011)	+	+	+	+	+	+	+	+	+
Darwood (2008)	+	-	-	-	+	+	+	+	+
Dwerryhouse (1999)	?	?	+	+	+	+	+	+	+
Eggen (2022)	+	?	-	-	+	+	+	+	+
Kalteis (2015)	?	?	?	+	+	+	+	+	+
Kempeneers (2022)	+	+	+	+	+	?	+	+	+
Lurie (2005)	+	+	+	+	+	+	+	+	+
Lam (2018)	+	+	-	+	+	+	+	+	+
Mohamed (2021)	+	+	-	-	+	+	+	+	+
Perala (2005)	+	+	?	+	+	+	+	+	?
Pronk (2010)	+	-	+	+	+	+	+	+	+
Rasmussen (2013)	+	+	+	?	+	+	+	+	+
Rass (2015)	+	+	?	?	+	+	+	+	?
Samuel	+	+	-	+	+	+	+	+	+

(2013)									
Stotter (2006)	+	?	?	?	+	+	+	+	+
Vahaaho (2017)	+	+	+	+	+	+	+	+	+
Van der Velden (2015)	+	+	+	+	+	+	+	+	+
Wallace (2018)	+	?	-	-	+	+	+	+	+
Yang (2013)	+	?	?	?	+	+	+	+	+
	Legend: "+" : Low risk of bias; "-" : High risk of bias; "?": Unclear risk of bias.								

<u>Appendix 1.</u>

Search Strategy

(leg varicosis (MESH) OR varicose vein* OR varice OR varicosities OR varicosis OR saphenous vein (MESH) OR saphenous vein) NOT (oesophagus varices (MESH) or Stomach varices (MESH))

AND

(Phlebectomy (MESH) OR Stripping OR ligation OR Laser surgery (MESH) OR EVLA OR EVLT OR sclerotherapy OR sclerotherapy OR endovenous laser OR avulsion OR VNUS OR mechanochemical ablation or MOCA OR ((thermal OR radio frequency OR laser) AND (ablation OR Obliteration))

AND

(recurrent disease (MESH) OR recurrent disease OR recurrence OR recurring OR recanalization OR recanali*ed OR re*opening OR reappearance OR reintervention OR repeat intervention OR recurrent OR neovascularization OR neovascularization (pathology) OR residual OR venous reflux OR vein* reflux OR GSV reflux OR SSV reflux OR AASV reflux OR saphenous vein* reflux)
